# Supplementary material for: Sex differences in the genetic regulation of the human plasma proteome
Source: Nat Commun. 2025 May 13;16:4001. doi: 10.1038/s41467-025-59034-4 (PMC12075630; doi:10.1038/s41467-025-59034-4)
Supplement: Supplementary file 1 — Supplementary Information [file 41467_2025_59034_MOESM1_ESM.pdf]

**Sex differences in the genetic regulation of the human plasma proteome**

**Supplementary Materials**

**Supplementary Figure 1: Comparison of effect size estimates of sex between the measurements from antibody-based and aptamer-based proteomics technologies for overlapping protein targets.** Linear regression models were used to test the association of sex with the protein abundance in each cohort. There were 1,991 unique protein target combinations between the two platforms targeting 1,838 unique proteins (defined by unique UniProt ID). Of these, findings for 1,006 were significant in both technologies (815 of which were also directionally consistent), 770 were significant in only one technology and 215 were not significant in any of these technologies. Overall, the correlation between the effect size estimates from the two technologies was r=0.60 for overlapping targets. The mean correlation (based on correlations by Eldjarn et al., Nature, 2023 (1)) for overlapping protein targets which were significant and directionally consistent in both technologies had a mean correlation coefficient (r) of 0.43 (SD=0.27), whereas the overlapping protein targets which were significant in both technologies but directionally inconsistent or were significant in only one technology had a correlation coefficient of 0.19 (SD=0.27) and 0.26 (SD=0.29), respectively.

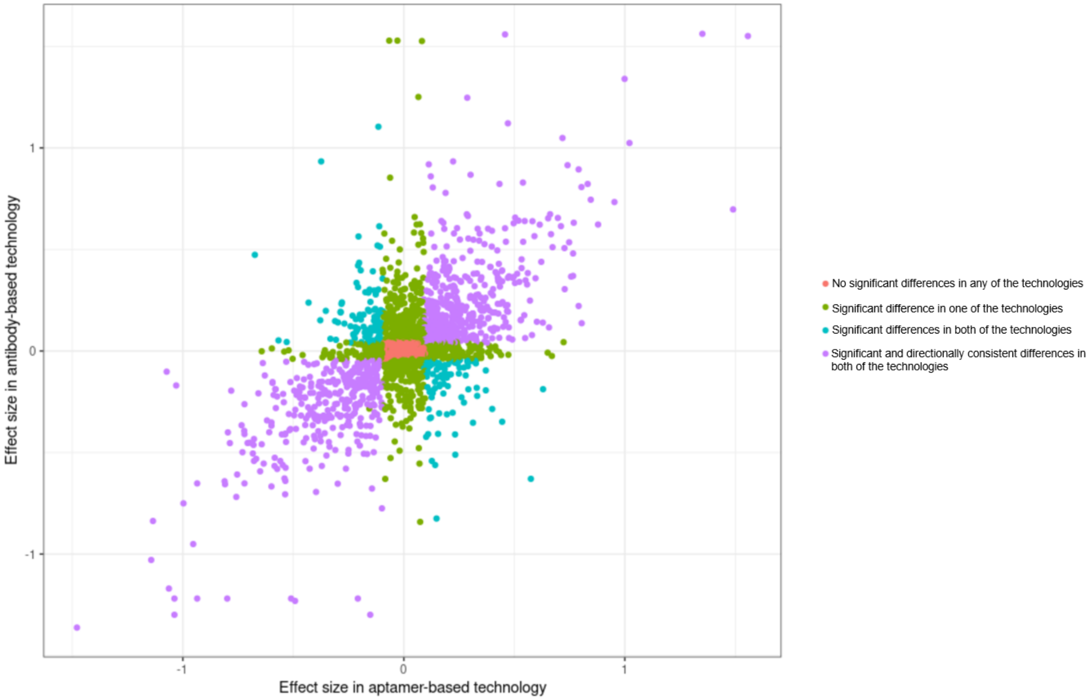

**Supplementary Figure 2: Summary of the study design and number of pQTLs identified.** Flowcharts with details of study design and number of pQTLs identified through genome-wide analyses of Fenland aptamer-based proteomic measurements (shown in purple) and UK Biobank antibody-based proteomic measurements (shown in orange).

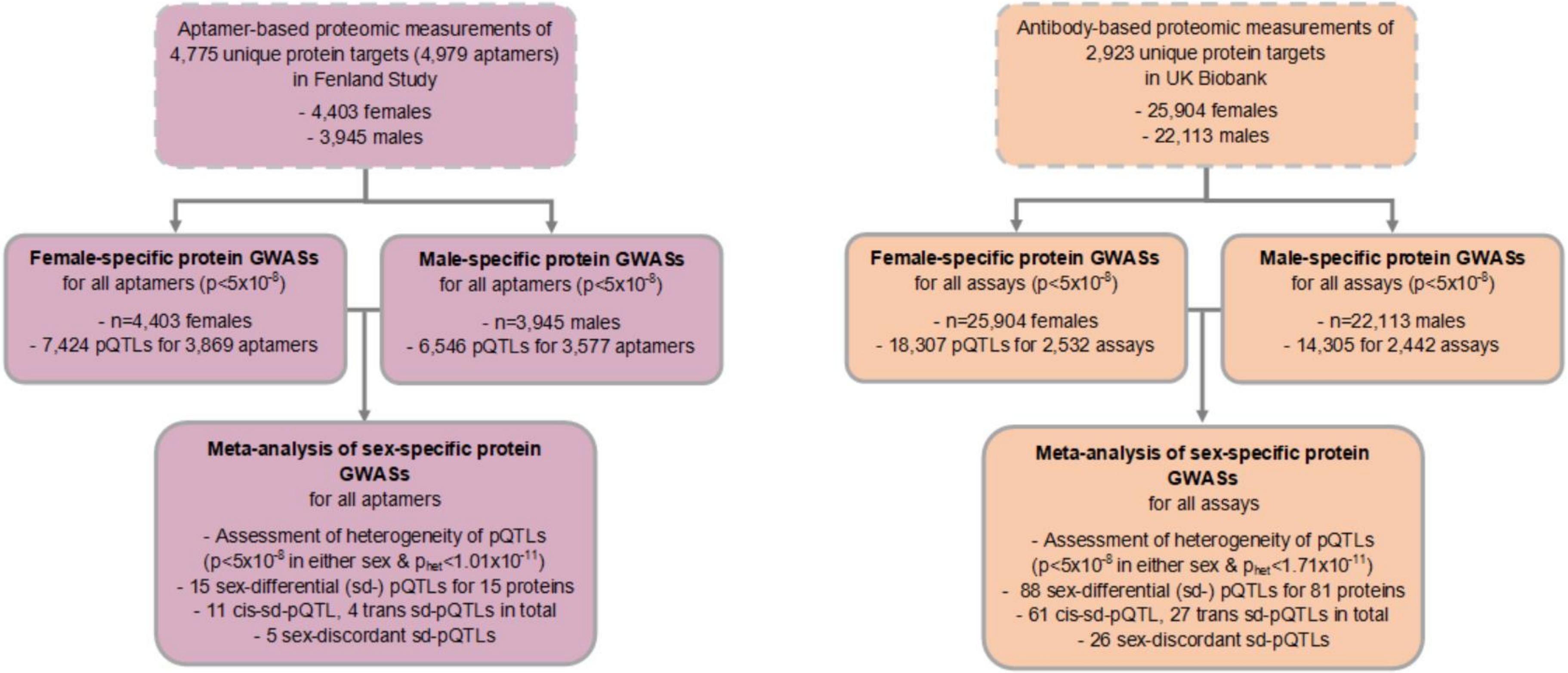

## References

1. Eldjarn GH, Ferkingstad E, Lund SH, Helgason H, Magnusson OT, Gunnarsdottir K, et al. Large-scale plasma proteomics comparisons through genetics and disease associations. Nature. 2023;622(7982):348-58.
2. Finan C, Gaulton A, Kruger FA, Lumbers RT, Shah T, Engmann J, et al. The druggable genome and support for target identification and validation in drug development. Sci Transl Med. 2017;9(383).
