# Supplementary material for: Sex differences in the genetic regulation of the human plasma proteome
Source: Nat Commun. 2025 May 13;16:4001. doi: 10.1038/s41467-025-59034-4 (PMC12075630; doi:10.1038/s41467-025-59034-4)
Supplement: Supplementary file 2 — Description of Additional Supplementary Datasets [file 41467_2025_59034_MOESM2_ESM.pdf]

Supplementary Datasets

Supplementary Data 1: Demographics of the Fenland and UK Biobank samples included in this study.

Supplementary Data 2. Observational sex-differences in protein abundances. To estimate the effect of sex, a linear regression model was for implemented in R 3.6, including covariates age and test site in the model in each platform in the model. The columns with "_bmi", "_ldl", "_alt", "_smoking" or "_alcohol" suffixes represent the results from sensitivity analyses where additional covariates were included as covariates in the model individually (BMI, LDL, ALT, smoking status and alcohol consumption, respectively). The columns with "_covars" suffix represent the results from sensitivity analyses where additional covariates were all included in the same model (BMI, LDL, ALT, smoking status and alcohol consumption were included). The columns with "_HRT_OC" suffix represent the results from sensitivity analyses where use of hormone replacement therapy/oral contraception were included as an additional covariate. "SL" and "OL" suffixes represents the results from aptamer- and antibody-based platforms, respectively. Information on draggability based on common gene entries from Finan et al. (2017) (2).

Supplementary Data 3. Sex-differences in the genetic regulation of protein abundances from aptamer-based technology in Fenland study. Linear regression models were used to identify protein quantitative trait loci (pQTLs) in each sex. This list includes all sd-pQTLs with a heterogeneity p-value<1.01e-11 between sexes from aptamer-based technology in Fenland study.

Supplementary Data 4. Sex-differences in the genetic regulation of protein abundances from antibody-based technology in UK Biobank study. Linear regression models were used to identify protein quantitative trait loci (pQTLs) in each sex. This list includes all sd-pQTLs with a heterogeneity p-value<1.71e-11 between sexes from antibody-based technology in UK Biobank study.

Supplementary Data 5. Phenome-wide association study results for 15 aptamer-based sd-pQTL variants using 365 binary phenotypes with >2,500 cases in UK Biobank. Logistic regression models were used to association between the variants and disease outcomes.

Supplementary Data 6. Phenome-wide association study results for 86 antibody-based sd-pQTL variants using 365 binary phenotypes with >2,500 cases in UK Biobank. Logistic regression models were used to association between the variants and disease outcomes.
